# Combined application of pharyngeal volume and minimal cross-sectional area may be helpful in screening persons suspected of obstructive sleep apnea (OSA)

**DOI:** 10.1007/s11325-021-02358-4

**Published:** 2021-05-08

**Authors:** Yuliang Zhao, Xinyu Li, Jiangang Ma

**Affiliations:** Department of Otolaryngology, The second hospital of Hebei Medical University, Shijiazhuang, 050000 China

**Keywords:** Obstructive sleep apnea (OSA), Acoustic pharyngometry (APh), Receiver operating characteristic (ROC) curve, Minimum cross-sectional area of pharyngeal cavity (mCSA), Pharyngeal volume

## Abstract

**Background:**

Obstructive sleep apnea (OSA) is a common disease that seriously affects human health and daily life. However, the gold standard for its diagnosis, polysomnography (PSG), is expensive resulting in inadequate diagnosis of this disease in primary clinics. Therefore, a simple and rapid method for initial screening for OSA is needed. Acoustic pharyngometry (APh) is an FDA-approved noninvasive method that is gradually being applied to screening for OSA.

**Materials And Methods:**

In this study, we applied analysis with receiver operating characteristic (ROC) curves to explore how APh may play a greater role in the screening of subjects with suspected OSA. Patients admitted into the departments of otolaryngology at our hospital from March 2017 to May 2019 were recruited into the study. All subjects underwent PSG monitor and were separated into two groups according to the apnea-hypopnea index (AHI) from the PSG results: OSA group (AHI ≥ 5) and control group (AHI < 5). APh measurements and other indicators of the subjects, including age, height, and weight; Epworth Sleepiness Scale (ESS) score; and the pharynx examination, including the degree of tonsil enlargement and tongue hypertrophy, were also be recorded.

**Results:**

The *t*-test results showed that almost all indicators except age and height have significant differences between the OSA group and control group. Subjects with OSA had greater weight, BMI, ESS, higher degree of tonsil enlargement, and tongue hypertrophy, while they had smaller minimal cross-sectional area (mCSA) and pharyngeal volume than the subjects in control group. The correlation analysis revealed that pharyngeal volume and mCSA were two helpful indicators to screen for OSA. Furthermore, we established the ROC curve and calculated the combining predictors (combining predictors = pharyngeal volume + mCSA * (− 2.347)/(− 0.225)). The area under the ROC curve (AUC) of combining predictors was 0.917 (95% CI 0.842–0.991, *P* < 0.001), which was higher than combinations of other two independent indicators. The cutoff point of combining predictors was found to be 59.84 (AUC = 0.917, sensitivity = 0.80, 1-specificity = 0.06, *P* < 0.001).

**Conclusions:**

These findings suggest that APh is a simple, rapid, and economical detection method which may be useful in screening for OSA, especially in communities and primary clinics where PSG cannot be performed.

## Background

Obstructive sleep apnea (OSA) refers to repeated episodes of apnea and hypopnea, hypercapnia, and sleep disruption, causing a series of clinical syndromes of pathophysiological changes [1]. Numerous studies have shown that OSA is associated with a variety of diseases, including hypertension [2]; coronary heart disease [3]; arrhythmia [4]; cerebrovascular disease [5]; type 2 diabetes [6]; nonalcoholic fatty liver disease [7]; kidney damage [8]; glaucoma [9]; sexual dysfunction [10]; and many other organ, multi-system damage. In order to effectively prevent the symptoms and these complications, a primary task is to screen for OSA in an early period.

In China, the diagnostic criteria for OSA was clearly defined at the 2002 Hangzhou Conference [11], and was revised in 2011 [12] and 2019 [13]. The current gold standard for the diagnosis and severity of OSA is overnight polysomnography (PSG). But the equipment for PSG monitoring is expensive and requires specialized venues and analysts. PSG cannot be universally utilized in community hospitals and primary clinics. Other examinations, such as Muller’s test lack simple and objective quantitative indicators for judging the location and severity of the obstruction. These factors have greatly limited the early diagnosis and treatment of OSA.

Acoustic pharyngometry (APh) is a FDA-approved, noninvasive method used in sleep apnea clinics and researches [14]. As an emerging technique for measuring the volume and cross-sectional area of the pharyngeal cavity by the principle of acoustic reflection, APh is characterized by simplicity, rapidity, and noninvasiveness, making it widely used in the preliminary screening of sleep apnea. Previous studies about the pharyngeal detection with APh were almost for normal people [14, 15], and a study exploring the relationship between APh and OSA did not clearly point out specific indicators and reference values [16].

Receiver operating characteristic (ROC) curve analysis is widely used as an estimate of the diagnostic value for many diseases. Based on our previous study on the application of APh [17, 18], we selected and measured the APh parameters of the subjects. Combined with PSG monitoring results, ESS score, and anthropometric variables, we tried to explore the upper airway anatomy of patients with OSA, and seek the correlations between APh parameters and the severity of OSA. The purpose of this investigation was to assess whether or not it is possible to screen for OSA through measurement of pharyngeal volume and minimal cross-sectional area with acoustic pharyngometry.We proposed to use ROC analysis, to provide a more accurate analysis.

## Materials and methods

### Protocol

This study selected person who were admitted to the Department of Otolaryngology in our hospital from May 2017 to May 2019 as subjects.

#### Inclusion criteria

A. Age 18–65 years old.

B. No gender limitation.

C. The patient was initially determined to be suspected of OSA by the chief physician.

#### Exclusion criteria

A. Age < 18 years old or > 65 years old.

B. Nasopharyngeal or oropharynx diseases and other malformations.

C. Severe cardiopulmonary disease.

D. History of acute upper respiratory tract infection in the past month: nasal congestion, sneezing, salivation, etc.

E. Did not obtain the consent of the subject.

All of the subjects underwent PSG monitoring and were subsequently separated into two groups according to the apnea-hypopnea index (AHI): OSA group (AHI ≥ 5) and control group (AHI < 5). The APh measurement and other indicators of the subjects, including age, height, and weight; ESS score; pharynx examination; degree of tonsil enlargement; and tongue hypertrophy, were also recorded soon afterwards.

This study was approved by the Research Ethics Committee of the Second Hospital of Hebei Medical University. The approval number is 2018-R251.

### General information

General information about the subjects such as gender and age was recorded. Height and weight of all subjects were measured and BMI was calculated (BMI = height/weight^2^). The subjects also completed the ESS. Physical examination of the pharyngeal cavity structures was performed, the degree of tonsil enlargement and tongue hypertrophy (0structure, d and which is recorded as 0, 1, 2, 3 points, were recorded as described in previous studies [19, 20].

### Polysomnography monitor

The Alice 4 polysomnography instrument (manufacturer: Compumedics; address: 30–40 Flockhart St. Abbotsford, Victoria, 3067, Australia) was used to perform night sleep monitoring for no less than 7 h. The monitoring items included electroencephalogram, electrocardiogram, electromyogram, electrooculogram, saturation of blood oxygen (SaO_2_), mouth and nasal airflow, chest and abdomen movement, position signal, and snoring. The results of the monitoring were analyzed by a single physician to obtain precise results. According to the criteria published by “Chinese Medicine Doctors Association Sleep medicine Specialized Committee (CMDASM) [13] and “American Academy of Sleep Medicine (AASM)” [21], “apnea” was defined as the nasal pressure signal 90% lower than the baseline value, with duration not less than 10 s. Hypopnea was scored when the nasal pressure signal was 50% lower than the baseline value, and the duration was not less than 10 s, accompanied by SaO_2_ decrease of 3%. AHI was calculated as the sum of the number of apneas and hypopneas that occurred per hour of sleep.

### Acoustic pharyngometry

An acoustic rhinometer instrument (manufacturer: GM INSTRUMENTS LTD; address: Unit 6, Ashgrove, Ashgrove Road Kilwinning, KA13 6PU, UK; name and version of the software: Acoustic, 3.2.0.1300) was used for APh measurement. All operations were performed by a single experienced physician. The measurement was carried out in a quiet room. The subject first assumed a sitting posture, tightened the sonic tube with the lips, and fixed it at the upper middle incisor to prevent air leakage keeping the sonic tube in a horizontal position (not tilted). The subject breathed through the nose 2 to 3 times and held his/her breath; then, he/she started the device for about 4–5 s. The sounder emitted 4 event waves, and the computer drew 4 curves. The measurement results were recorded in the form of a graph and obtained the area-distance curve that is the APh curve. The same method was used to measure the results in the supine posture and the lateral posture. Each operation was repeated 3 times, and the repetition rates were controlled within 10%. The average values were taken to obtain a series of parameter values.

The 0 cm of the abscissa of the APh curve corresponded to the upper middle incisor. The measurement range was 0–20 cm, and the observation range was about 7–17 cm, i.e. the oropharynx junction to the glottis area. The computer automatically calculated the volume of the area as the pharyngeal volume. According to the results of previous studies [17], the mCSA and the pharyngeal volume were selected as the parameters of APh in three postures (sitting posture, supine posture, and lateral posture).

### Statistical analyses

Data were analyzed using IBM SPSS Statistics 25 software package. Continuous variables were expressed as mean ± standard deviation. Student’s *t* test was used for continuous variables including age, height, weight, BMI, ESS score, AHI, LSaO2, SIT90, pharyngeal volume, and mCSA. Chi-square tests were used for discrete variables including gender. Kruskal-Wallis test was used for degree of tonsil enlargement and tongue hypertrophy. Correlation analysis was used to analyze the correlations between the pharyngeal volume and indicators of the PSG results (AHI, SIT90, and LSaO_2_) respectively. A *p* value < 0.05 was considered statistically significant. The ROC curve was established, the regression equation was obtained, the joint prediction factor was calculated, and the maximum Youden index as the cutoff point was selected. All illustrations were generated using Graph Prism 6.0 software.

## Result

A total of 68 subjects were studied. There were 52 subjects in the OSA group, including 35 men and 17 women. There were 16 subjects in the control (non-OSA) group, including 12 men and 4 women. Chi-square test showed no significant difference in sex ratio between the two groups (*χ*^2^ = 0.339, *P* = 0.560).

### Comparison of different indicators between OSA group and control group

The results show that (1) there were no significant differences in the gender, age, and height between the control group and the OSA group; (2) body weight, BMI, ESS score, AHI, SIT90, tonsil enlargement, and tongue hypertrophy in the OSA group were significantly greater than those in the control group; and (3) the LSaO_2_, pharyngeal volume, and mCSA in three postures in the OSA group were significantly lower than those of the control group. Data are shown in Tables 1 and 2.

**Table 1:** Comparison of different indicators between OSA group and control group

	Indicators	Control group	OSA group	*t*	*P*
General condition	Age	35.5 ± 2.6	37.4 ± 1.5	.6236	0.54
	Height (cm)	171.9 ± 1.6	170.0 ± 0.9	1.006	0.32
	Weight (kg)	68.8 ± 2.3	104.2 ± 3.0	6.245	*< 0.001*
	BMI	23.2 ± 0.6	36.0 ± 1.0	6.696	*< 0.001*
	ESS	1.9 ± 0.4	8.8 ± 0.8	4.587	*< 0.001*
PSG results	AHI (times/h)	0.6 ± 1.1	40.4 ± 4.6	4.419	*< 0.001*
	LSaO_2_ (%)	87 ± 2	72 ± 2	3.598	*0.006*
	SIT90	0.36 ± 0.24	23.14 ± 3.56	3.527	*0.008*
	Pharyngeal volume (cm^3^)	48.6 ± 1.2	35.0 ± 1.1	6.650	*< 0.001*
APh results	mCSA (supine) (cm^2^)	1.77 ± 0.06	1.29 ± 0.05	4.786	*< 0.001*
	mCSA (sitting) (cm^2^)	2.03 ± 0.11	1.70 ± 0.06	2.614	*0.011*
	mCSA (lateral) (cm^2^)	1.68 ± 0.11	1.43 ± 0.05	2.300	*0.025*

**Table 2:** Kruskal-Wallis test for the degree of tonsil enlargement and tongue hypertrophy

Indicators	Mean rank		*χ*^2^	*P*
	Control group	OSA group		
Tonsil enlargement	13.13	41.08	26.682	*< 0.001*
Tongue hypertrophy	19.25	39.19	13.350	*< 0.001*

### Correlation analysis between APh results and PSG

There were significant correlations between the pharyngeal volume vs. all indicators of the PSG results (*P <* 0.001). However, the correlations between the mCSA in the three postures vs. the PSG results were not exactly the same: There were significant correlations between the mCSA in the supine position vs. the indicators of the PSG results (*P <* 0.001, *P =* 0.008, *P =* 0.002); in the lateral posture, there were significant correlations between the mCSA vs. AHI (*P =* 0.006), while there was no significance vs. the other indicators; there was no significant correlation between the mCSA in the sitting vs. the indicators of the PSG results. Data shown are in Table 3.

**Table 3:** Correlation analysis between APh results vs. PSG results

Indicators		AHI	SIT90	LSaO_2_	
Pharyngeal volume	Pearson correlation	*− .690*	*− .502*	*.555*	
	Sig. (two-tailed)	*< .001*	*< .001*	*< .001*	
mCSA (sitting)	Pearson correlation	**−** .231	**−** .120	.095	
	Sig. (two-tailed)	.058	.328	.440	
mCSA (supine)	Pearson correlation	*− .525*	*− .321*	*.372*	
	Sig. (two-tailed)	*< .001*	*.008*	*.002*	
mCSA (lateral)	Pearson correlation	*− .328*	**−** .206	.183	
	Sig. (two-tailed)	*.006*	.091	.135	

### Established the ROC curve and acquired cutoff point

According to the results above, we found that APh measurement of pharyngeal volume and mCSA in supine position were two favorable indicators for the screening of OSA. Next, we established the ROC curve to determine the effectiveness of these two indicators. The areas under the ROC curve (AUC) of pharyngeal volume and mCSA were 0.887 (95% CI 0.789–0.985, *P* < 0.001) and 0.828 (95% CI 0.731–0.925, *P* < 0.001). Then, we calculated the combining predictors (a new indicator obtained by logistic regression analysis).

The regression equation was:

$$ \mathrm{Logit}\ (Y)=13.750+\left(-0.225\right)\ast \mathrm{pharyngeal}\ \mathrm{volume}+\left(-2.347\right)\ast \mathrm{mCSA}. $$

And the combining predictors were calculated as:

$$ \mathrm{Combining}\ \mathrm{predictors}=\mathrm{pharyngeal}\ \mathrm{volume}+\mathrm{mCSA}\ast \left(-2.347\right)/\left(-0.225\right). $$

**Fig. 1:**
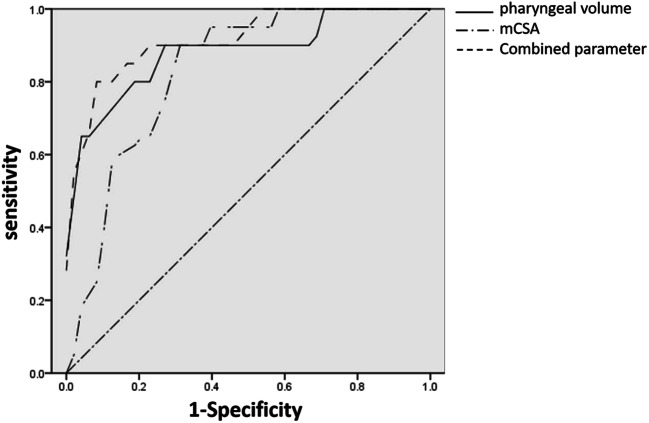
The ROC curve of pharyngeal volume, mCSA, and combining predictors

The AUC of combining predictors was 0.917 (95% CI 0.842–0.991, *P* < 0.001), which was higher than other two independent indicators. The cutoff point of combining predictors was received as 59.84 (AUC = 0.917, sensitivity = 0.80, 1-specificity = 0.06, *P* < 0.001) (Fig.1).

## Discussion

### The primary general survey of OSA is imminent

OSA is characterized by repeated episodes of apnea and hypopnea, hypercapnia, and sleep disruption. According to the World Health Organization, about 100 million people worldwide suffer from OSA [22]. A meta-analysis of OSA epidemiological studies [2] pointed out that the morbidity of OSA in the general adult population was 9–38% worldwide and men’s were slightly higher than women’s. This proportion increases with age. In some elderly populations, the morbidity rate was as high as 90% for men and 78% for women [20]. Some cross-sectional studies had reported a morbidity of OSA ranging from 40 to 90% in individuals with a BMI > 40 kg/m^2^ (severe obesity) [23].

Although the known morbidity is high, the factual situation may be more serious. Young et al. estimated that up to 93% of women and 82% of moderate OSA patients have not been diagnosed; as many as one in five adults may have been mild undiagnosed OSA; more than one-tenth adults may had moderate undiagnosed disease [24–26]. The results of a selective screening study were startling: In the group only 19% of patients were previously diagnosed with OSA, but the diagnostic rate increased to 56% after being screened by clinical parameters and the ESS score. Furthermore, when all patients were examined by PSG, the diagnostic rate increased to 91% [27]. The underestimation of OSA diagnosis in the general population means there had been a large number of undiagnosed OSA patients who were mistaken for non-OSA patients. In terms of treatment, in the Sleep Heart Health Study of more than 15,000 people, the morbidity rate of OSA was more than 4%, while only 1.6% of patients were diagnosed by doctors, and only 0.6% were actually treated [28]. These results indicated that the diagnosis of this disease was seriously inadequate, and the general population has insufficient understanding of the severity of the disease and lack of willingness to be treated. Therefore, focusing on high-risk factors, getting patients identified sooner, and starting intervention sooner were important approaches to reducing the harms of OSA. The major reason for underdiagnosis is the limited diagnostic method. The gold standard is PSG, but the equipment is expensive and requires specialized venues and analysts. There are many other diagnostic methods for OSA described in the literature, but each has limitations. Neck circumference is easy to measure but underestimates OSA in lean individuals [29], and does not perform as well as pharyngometry. CT involves ionizing radiation exposure and is not readily available in many sleep clinics or other offsite centers. CT is also time-consuming and costly [30]. MRI avoids ionizing radiation and provides excellent definition of parapharyngeal soft tissues [31], but is expensive and not readily available in many sleep clinics. ESS was often used for the initial screening of OSA, but because of its subjectivity, the study of the effectiveness and accuracy of the scale had different conclusions [32]. Other examinations such as Muller’s test always lack simple and objective quantitative indicators for judging the location and severity of obstruction. These factors have greatly limited the early diagnosis and treatment of OSA. Therefore, we urgently need a simple, convenient, and economical method for screening OSA which can be used in clinics.

### APh can be used for initial screening of OSA

As an emerging technique for measuring the volume and cross-sectional area of the pharyngeal cavity by the principle of acoustic reflection, APh is an FDA-approved noninvasive diagnostic test that is gradually gaining use in screening for OSA. The APh is a simple instrument yielding rapid results.

**Fig. 2:**
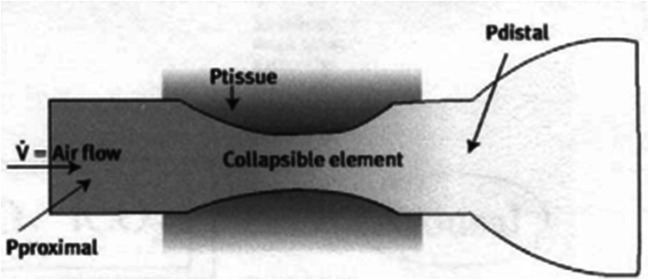
Starling resistor model [24]

In patients with OSA there is the narrowing or blocking of the upper respiratory tract from the oropharynx to the hypopharynx during sleep, resulting in ineffective or absent breathing. Accurate understanding of the upper respiratory tract anatomy is the key to screening for OSA. The Starling resistor model has been proposed to explain the complex series of events that occur in patients with OSA during sleep (Fig. 2) [24]. Pharyngeal size, compliance, and the dynamic behavior of the upper airway have been considered important factors in the pathogenesis of OSA. So, the assessment of the precise narrowing site of the upper airway may not be only one of the keys in understanding the pathogenesis of this disorder but also in improving the management of this condition. APh has the potential to be a useful tool for localizing the possible site(s) of upper airway obstruction in cases of OSA.

In 1984, Rilive et al. [33] first applied APh to patients with OSA. By measuring the upper respiratory tract morphology, it was concluded that the average cross-sectional area of the pharyngeal cavity and the average glottal cross-sectional area in OSA was smaller than in patients without OSA. Kamal et al. [15] used special techniques during the examination to locate the oropharyngeal junction and the glottis to generate a mapped acoustic throat map, calculating the minimum pharyngeal area and glottal area. The studies’ results showed that the APh can accurately determine the pharyngeal obstruction [14], and had been validated by computerized axial tomography (CT) [34] and magnetic resonance imaging (MRI) [35].

The APh results showed good repeatability and a low degree of variability in the measurement of the oral cavity and pharyngeal cavity [36]. In particular, mCSA obtained via APh has been established to have acceptable intrasession and test-retest reliability [15].

### Combined application of pharyngeal volume and mCSA can be a favorable and helpful indicator of OSA screening

The results of our study showed that subjects with OSA had higher weight and BMI than subjects with no OSA. Obesity has been considered to be a major risk factor for OSA and was closely related to the severity of OSA [37]. The upper airway structure of obese people is significantly different from normal people. With increased body fat and reduced lung volume (functional residual capacity), the upper airway in obese patients is narrower, and makes the airway vulnerable to collapse. Obesity can also lead to increased fat deposition around the pharyngeal wall and lymphoid tissue proliferation in the neck. The fat deposition in the pharynx has an external compression effect, which causes the pharyngeal cavity to change from a normal circular shape to an elliptical shape resulting in a reduction in the upper airway cross-sectional area and an increase in airflow resistance. Parts of the upper airway narrow (including nasal cavity, nasopharynx, oropharynx, and throat) affecting normal breathing [38]. Previous studies have shown that gender, age, height, and other factors affect the pharyngeal cavity structure (mainly pharyngeal volume) [39], and the mCSA decrease with age [40]. Examination of the upper airway has shown that subjects with OSA have more tonsil enlargement and tongue hypertrophy than controls. Gun’s study showed that the cross-sectional area and volume of the upper airway is smaller in the supine position than any other positions and is the most predictive parameter to discriminate between subjects with or without OSA [41]. Another study demonstrated that Aph measurement of mCSA is a significant independent predictor of moderate-to-severe obstructive sleep apnea [42], which is consistent with our study. In all, a variety of factors such as obesity, inflammation, and metabolic disorders may cause complex changes in the structure of the pharyngeal cavity and lead to OSA.

### Applied ROC curve to conduct a further analysis of the APH results

The main purpose of our study was to find the appropriate parameters using APh for indicating OSA. Based on previous studies [15] and the results of our investigation, we have found that pharyngeal volume and mCSA are useful to screen for suspected OSA when PSG is not available. According to the ROC curve analysis, combining predictors had the largest AUC. The findings of this research confirms that APh is a simple and rapid method to screen for OSA. Aph may be especially pertinent for use in communities and primary clinics where PSG cannot be performed.

## Conclusion

The current study provides evidence that APh may play a role in screening for OSA. Pharyngeal volume and mCSA appear to be helpful indicators suggesting the presence of OSA. If combining predictors (calculated as: combining predictors = pharyngeal volume + mCSA * (− 2.347)/(− 0.225)) show a score higher than 59.84, OSA is very likely (sensitivity = 0.80, 1-specificity = 0.06) and suggests the need for further examination with PSG.

## Data Availability

All data generated or analyzed during this study are included in this published article. The database during and/or analyzed during the current study available from the corresponding author on reasonable request.

## Abbreviations

OSA: obstructive sleep apnea
APh: acoustic pharyngometry
ROC: receiver operating characteristic
ESS: Epworth Sleepiness Scale
mCSA: minimal cross-sectional area
PSG: polysomnography
SaO_2_: saturation of blood oxygen
AHI: apnea and hypopnea index
LSaO_2_: lowest saturation of blood oxygen
SIT90%: salvation impair time below 90%
CT: computerized axial tomography
MRI: magnetic resonance imaging
